# Bioreactor-Grown Bacillus of Calmette and Guérin (BCG) Vaccine Protects Badgers against Virulent *Mycobacterium bovis* When Administered Orally: Identifying Limitations in Baited Vaccine Delivery

**DOI:** 10.3390/pharmaceutics12080782

**Published:** 2020-08-18

**Authors:** Sandrine Lesellier, Colin P. D. Birch, Dipesh Davé, Deanna Dalley, Sonya Gowtage, Simonette Palmer, Claire McKenna, Gareth A. Williams, Roland Ashford, Ute Weyer, Sarah Beatham, Julia Coats, Alex Nunez, Pedro Sanchez-Cordon, John Spiropoulos, Stephen Powell, Jason Sawyer, Jordan Pascoe, Charlotte Hendon-Dunn, Joanna Bacon, Mark A. Chambers

**Affiliations:** 1 Department of Bacteriology, Animal and Plant Health Agency, Woodham Lane, New Haw, Addlestone, Surrey KT15 3NB, UK; Sandrine.lesellier@anses.fr (S.L.); Dipesh.Dave@apha.gov.uk (D.D.); deanna.dalley@apha.gov.uk (D.D.); Sonya.Gowtage@apha.gov.uk (S.G.); Si.Palmer@apha.gov.uk (S.P.); claire.mckenna@apha.gov.uk (C.M.); Gareth.A.Williams@apha.gov.uk (G.A.W.); Roland.Ashford@apha.gov.uk (R.A.); jason.sawyer@apha.gov.uk (J.S.); 2 Laboratoire de la Rage et de la Faune Sauvage de Nancy (LRFSN), Technopole Agricole et Vétérinaire, Domaine de Pixérécourt-Bât. H., CS 40009-54220 Malzéville, France; 3 Department of Epidemiological Sciences, Animal and Plant Health Agency, Woodham Lane, New Haw, Addlestone, Surrey KT15 3NB, UK; Colin.Birch@apha.gov.uk; 4 Animal Services Unit, Animal and Plant Health Agency, Woodham Lane, New Haw, Addlestone, Surrey KT15 3NB, UK; ute.weyer@apha.gov.uk; 5 Animal and Plant Health Agency, Sand Hutton Campus, York, North Yorkshire YO41 1LZ, UK; Sarah.Beatham@apha.gov.uk (S.B.); Julia.Coats@apha.gov.uk (J.C.); 6 Department of Pathology, Animal and Plant Health Agency, Woodham Lane, New Haw, Addlestone, Surrey KT15 3NB, UK; Alejandro.Nunez@apha.gov.uk (A.N.); Pedro.Sanchez-Cordon@apha.gov.uk (P.S.-C.); John.Spiropoulos@apha.gov.uk (J.S.); 7 Data Systems Group, Animal and Plant Health Agency, Woodham Lane, New Haw, Addlestone, Surrey KT15 3NB, UK; Stephen.Powell@apha.gov.uk; 8 Public Health England, National Infection Service, Porton Down, Salisbury, Wiltshire SP4 0JG, UK; Jordan.Pascoe@phe.gov.uk (J.P.); Charlotte.Dunn@phe.gov.uk (C.H.-D.); Joanna.Bacon@phe.gov.uk (J.B.); 9 Faculty of Health and Medical Sciences, University of Surrey, Guildford, Surrey GU2 7XH, UK

**Keywords:** BCG, oral vaccine, tuberculosis, badgers, bait, fermentation

## Abstract

Bovine tuberculosis (TB) in Great Britain adversely affects animal health and welfare and is a cause of considerable economic loss. The situation is exacerbated by European badgers (*Meles meles*) acting as a wildlife source of recurrent *Mycobacterium bovis* infection to cattle. Vaccination of badgers against TB is a possible means to reduce and control bovine TB. The delivery of vaccine in oral bait holds the best prospect for vaccinating badgers over a wide geographical area. There are practical limitations over the volume and concentration of Bacillus of Calmette and Guérin (BCG) that can be prepared for inclusion in bait. The production of BCG in a bioreactor may overcome these issues. We evaluated the efficacy of oral, bioreactor-grown BCG against experimental TB in badgers. We demonstrated repeatable protection through the direct administration of at least 2.0 × 10^8^ colony forming units of BCG to the oral cavity, whereas vaccination via voluntary consumption of bait containing the same preparation of BCG did not result in demonstrable protection at the group-level, although a minority of badgers consuming bait showed immunological responses and protection after challenge equivalent to badgers receiving oral vaccine by direct administration. The need to deliver oral BCG in the context of a palatable and environmentally robust bait appears to introduce such variation in BCG delivery to sites of immune induction in the badger as to render experimental studies variable and inconsistent.

## 1. Introduction

Bovine tuberculosis (TB) in Great Britain adversely affects animal health and welfare and is a cause of considerable economic loss to farmers and Government [1]. Although transmission of the bacterium, *Mycobacterium bovis* between cattle is an important factor in spread of the disease [2], the European badger (*Meles meles*) represents an additional wildlife source of recurrent *M. bovis* infection to cattle in both the United Kingdom (UK) and Ireland [3]. Vaccination of badgers against TB is a possible means to reduce and control bovine TB alongside other control measures, including the vaccination of cattle in the future. Delivery of vaccine to badgers in oral bait holds the best prospect for vaccinating badgers over a wide geographical area [4] and has proved highly successful for mass vaccination of other wildlife species against rabies and classical swine fever in mainland Europe [5,6]. Success is dependent on combining a vaccine that is safe and efficacious via the oral route with attractive bait that can be delivered using a pragmatic and economical deployment protocol. Several studies report the successful development of an oral bait for use with wild badgers that is both attractive and highly palatable, as well as being compatible with the live, attenuated Bacillus of Calmette and Guérin (BCG) vaccine [7,8,9]. The vaccine itself has been shown to be efficacious when given orally to badgers, followed by experimental challenge with virulent *M. bovis* [10,11].

Conventional production of BCG is based on growth as a surface pellicle on liquid medium, followed by harvesting and ball-milling to generate a homogeneous suspension of live bacteria [12]. Although BCG licensed for parenteral administration to humans and badgers is produced this way, there is increasing interest in the possibility of producing the BCG vaccine through cultivation in bioreactors [12,13]. This approach has several advantages over conventional production, particularly the prospect of rapid, reproducible vaccine production under defined and strictly controlled conditions. The success of oral BCG vaccination in experimental challenge studies appears to be highly dependent on the dose of BCG administered but there is limitation on the concentration of BCG that can be prepared for inclusion in the candidate bait developed in the UK [8]. This is the result of constraint on the volume of BCG that can be incorporated in the candidate bait, together with the relatively high number of viable BCG needed to confer reliable protection when given orally. For these reasons, a further attraction of growing BCG in bioreactors for our context is the possibility of more cost-effective production of BCG at volumes and concentrations that exceed what is possible through pellicle growth.

Changing the method of vaccine preparation may introduce changes to the properties of the vaccine, including immunogenicity and reactogenicity [14], although these differences may be very slight [13]. We were interested therefore in the efficacy of BCG produced in a bioreactor as an oral preparation for vaccination of badgers against TB. Across two studies, we demonstrated reproducible protection achieved through the direct administration of at least 2.0 × 10^8^ colony forming units (CFU) of bioreactor-grown BCG Danish to the oral cavity of badgers, followed thirteen weeks later by endobronchial challenge with *M. bovis*. In contrast, vaccination via voluntary consumption of bait containing the same preparation of BCG did not result in demonstrable protection at the group-level. At the level of the individual animal however, we saw that where immunity was adequately induced there was subsequent protection from challenge. The need to deliver oral BCG in the context of a palatable and environmentally robust bait appears to introduce such variation in BCG delivery to sites of immune induction in the badger as to render experimental studies variable and inconsistent.

## 2. Materials and Methods

### 2.1. Reagents

Ingredients of Middlebrook 7H9 (TSE compliant): 7H9 broth base (BD Difco, Wokingham, UK, Cat no: 271310), 10% *v/v* OADC supplement (BD BBL, BD Difco, Wokingham, UK, Cat no: 212240), 0.5% *v/v* Glycerol (VWR International, Radnor, USA, Cat no: 49779-1 L), 0.2% *v/v* Tween 80 (Merck Life Science UK Limited, Gillingham, UK, Cat no: P6224-500 mL). The medium was prepared as follows: 4.7 g of broth base was added to 900 mL water along with 5 mL Glycerol and 2 mL Tween80. The broth was autoclaved at 124 °C for 20 min, being allowed to cool prior to adding 100 mL OADC.

### 2.2. Preparation of Working Seed

The inoculum used for generation of a working seed was prepared as follows: four plates of Middlebrook 7H10 were spread with *M. bovis* BCG from commercial vaccine stock (Danish 1331, AJ Vaccines, Copenhagen, Denmark) and incubated for four weeks. The biomass from two of these agar plates was re-suspended in 20 mL of sterile water and used as the inoculum for bioreactor-growth in 2 L of Middlebrook 7H9 medium (as described below). For preparation of the working seed the culture was harvested by centrifugation at 4600× *g* for 20 min. The cell pellet was re-suspended in 0.1× culture volume of 1.5% monosodium glutamate (MSG; *w/v* in water). The re-suspended culture was aliquoted in volumes of 1 mL and stored at −80 °C.

### 2.3. Bioreactor-Growth of BCG Vaccine

The inoculum used for growing batches of BCG for vaccination were prepared as follows: Middlebrook 7H10 agar plates were spread with the working seed. These were incubated at 37 °C for three weeks. A loopful of growth was used to inoculate 50 mL of Middlebrook 7H9 in five 125 mL Erlenmeyer vented flasks. These were incubated for four days at 37 °C whilst shaking at 200 rpm. The five BCG cultures were pooled and adjusted to an optical density (OD600 nm) of 1.0 to give a total volume of 250 mL of bacilli. Each batch of BCG vaccine consisted of three independent bioreactor cultures, grown in Middlebrook 7H9. A large bioreactor (2 L capacity), was inoculated with 100 mL of inoculum and two smaller bioreactors (0.8 L capacity) were inoculated with 40 mL of inoculum; equating to a 5% *v/v* of the culture volume. The cultures were agitated by a magnetic bar placed in the culture vessel coupled to a magnetic stirrer positioned beneath the vessel. Culture conditions were continuously monitored by an Eycoferm Fermentation System (Brighton Systems, Newhaven), linked to sensor probes inserted into the culture through sealed ports in the top plate. Culture temperatures were monitored using a dual wire stainless steel temperature probe and maintained at 37 °C by a heating pad positioned beneath the culture vessel. The cultures were stirred at an agitation rate of 500 to 750 rpm. The oxygen concentration was monitored with a galvanic oxygen electrode (Broadley-James Ltd., Bedford, UK) and the air saturation was maintained at 50% (10% dissolved oxygen tension). The initial pH of the cultures was set at 6.7 and was monitored throughout the experiment using an Ingold pH electrode (Mettler Toledo, Leicester, UK). All parameters were controlled and logged via the Eycoferm controller. Each culture was maintained for ~7 days, at which point they were harvested.

#### Harvest of BCG

For the harvest of bioreactor-grown BCG for vaccination, cultures were pooled and spun at 4600× *g* for 20 min. The supernatant was decanted, and the pellets were re suspended and pooled in approximately 100 mL of 1.5% monosodium glutamate (MSG) (*w/v* in water). The supernatant was spun for a second time at 4600× *g* for 20 min. The supernatant was discarded, and the pellets were re-suspended in approximately 20 mL 1.5% MSG before being added to the previous re-suspended pellets. To the cell suspension 1.5% MSG was added to a final volume that was equivalent to 10% of the original culture volume. The BCG suspension was stored at 4 °C.

### 2.4. Mycobacterium bovis

The *M. bovis* strain used for challenge was originally isolated from a naturally infected badger in the UK in 1997 (isolate 74/0449/97). All vials of the challenge strain are stored at the Animal and Plant Health Agency (APHA) at −80 °C until use at concentration range of 10^7^ to 10^8^ CFU/mL. On the day before challenge, one aliquot of the challenge strain was thawed, and submitted to serial dilution in sterile water +0.05% tween 80 to obtain approximately 5 × 10^3^ CFU/mL. The dilutions were vortexed to diminish the risk of bacterial clumping. The final dilution (challenge inoculum) was made into sterile phosphate-buffered saline (PBS) + 0.05% tween 80 and placed inside 3 mL syringes on the day prior to the first challenge and kept at +4 °C until all the animals were challenged over the course of four days (with one control group challenged on each day). Once each day, the dose of *M. bovis* contained in a syringe was measured retrospectively on three, individual Modified Middlebrook 7H11 culture plates (100 µL inoculum per plate) placed at 37 °C for four weeks.

### 2.5. Badgers

Forty adult badgers were enrolled in each of two experiments (referred to as Vaccine Efficacy Study (VES)9 and VES10, respectively). The animals were trapped from a British county (Northumberland) with no reported cases of TB in badgers or cattle and from TB-free areas of two other British counties (Yorkshire and Surrey), under the terms of a Natural England licence. Also included were badgers born in captivity at APHA from TB-free badgers. Wild, trapped animals were confirmed TB-free during a quarantine period when a series of three Interferon Gamma Release Assays (IGRA) [15] were conducted at approximately one-month intervals. Clinical samples (tracheal mucus and rectal swabs) were negative by culture for *M. bovis* just after the start of each study. All experimental procedures underwent ethical approval by the establishment and were authorised under Home Office project licenses PPL70/7878 and P25FCD7D8 with Home Office approved end-points referring to the welfare of badgers in captivity. No clinical sign associated with experimental TB infection was expected nor observed. All procedures (except the delivery of oral vaccine baits which were consumed voluntarily) required a general anaesthesia with a cocktail of ketamine, butorphenol and medetomidine injected intramuscularly and occasional top ups with isoflurane as described previously [16]. Each animal was uniquely identified by a subcutaneous microchip and by clip marking to recognize the animal during infra-red (IR) observation by camera. The animals were housed in captivity in stable social groups of four animals per pen in an outdoor facility until four to six weeks before experimental challenge when they were then moved and housed with the same social group to individual rooms within an Advisory Committee on Dangerous Pathogens (ACDP) Containment Level 3 (CL3) facility where they were challenged with *M. bovis* to test the protective efficacy of BCG. This period was planned to allow them to acclimatise to their new environment. In each facility, individual pens held at least one large wooden box which the badgers could access freely and use as a group sett. Between facilities, the badgers were transported conscious, secured inside their wooden setts. Objects, such as concrete tunnels and wooden objects were placed in the pens as environmental enrichment. Throughout each study, the badgers received a diet of dog food (moist and dry) and peanuts. Some of the food was hidden or scattered in the pen to encourage the exploratory behaviour of the badgers. Occasionally they were given eggs. Tap water was available ad libitum in large trays allowing both bathing and drinking. The badgers were observed daily by trained staff members after opening the wooden setts, and regularly using infra-red cameras, in particular after the badgers’ initial trapping, transport to CL3 building, and after vaccination and challenge. The animals remained in good conditions over the course of the two studies, except for two animals (both in the manual vaccination group of VES10, therefore in different pens and social groups), which were removed for welfare reasons (unexplained weight loss and a non-treatable wound, respectively) before experimental challenge.

At regular intervals and under general anaesthesia, blood was collected into Vacutainer^®^ serum-separating tubes, or those containing heparin, and occasionally ethylenediaminetetraacetic acid, EDTA, as well as clinical samples (tracheal aspirates, laryngeal swabs and rectal swabs), which were submitted for BCG/*M. bovis* culture. On these occasions, the clinical condition of each animal was recorded.

### 2.6. Vaccination

Badgers were vaccinated with *Mycobacterium bovis* BCG Danish strain 1331 in 1.5% (*w/v*) monosodium glutamate that had been grown in a bioreactor, harvested and concentrated by centrifugation (4600× *g*, 20 min). Each animal within a social group was randomly allocated to a treatment, so that each of the ten pens contained one animal allocated to the control treatment, and the remaining three animals allocated to receive vaccine either manually under general anaesthesia (VES9 and VES10 studies) or by voluntary consumption of BCG containing baits (in VES10 only). The manual vaccination doses of BCG were prepared in sterile conical-bottomed polypropylene microcentrifuge tubes. The voluntary consumption vaccination doses were prepared by incorporating BCG into a cavity within the badger baits, produced as previously described [8].

All vaccine doses, including those in baits, were stored at 2–8 °C until the time of vaccination: VES9, 34–35 days’ storage and VES10, 76–84 days’ storage. The manual vaccination of badgers (20 animals in VES9 and ten in VES10) was conducted under generalised anaesthesia over a single day for each study. Each badger received 200 μL BCG, administered using a micropipette as follows: 50 μL approximately 2 mm above each of the left and right pharyngeal tonsils allowing the suspension to percolate over the tonsils and 50 μL under the tongue on each of the left and right sides. After vaccination, badgers were monitored closely for approximately five minutes for coughing or signs of discomfort (neither observed) and to prevent liquid drainage from the mouth, then returned to their wooden setts to recover from general anaesthesia. In VES10, 20 animals each received a fresh single BCG bait packaged in a paper bag (approx. 145 × 75 mm) with thin grease-proof lining (Ref YPBFERAL, York Paper Company, Auckland, New Zealand; supplied by Connovation Ltd., Auckland, New Zealand). Each bag had two holes punched in its top half to assist with the release of odour cues to encourage consumption. An additional ten (control) badgers each received a placebo bait prepared and presented in the same way as for vaccine bait but containing no BCG. Bait was placed in individual cages, each containing a single badger, on up to three separate nights if not consumed on the previous occasions. The badger was released from its holding cage each morning. To increase the chances of consumption, the badgers had been habituated to consume packaged baits (without BCG) before the start of the study.

The BCG vaccination doses administered to each treatment group were determined using samples of the vaccines that had been exposed to the same environmental conditions as the administered vaccines. As vaccination in each of the studies took place over several days, BCG doses were determined on each vaccination day by titration using Sauton-Tween 80 medium as a diluent and by culture on Modified Middlebrook 7H11 agar at 37 °C for 4–5 weeks.

### 2.7. Challenge

Whilst under general anaesthesia, the badgers received 1.35–2.57 × 10^3^ or 1.21–2.50 × 10^3^ CFU in 1 mL solution in VES9 and VES10, respectively according to the model of endobronchial infection with *M. bovis* developed and reported previously [17]. In this model, the bronchial entrance of the right middle lobe was targeted with an endoscope (Olympus fibrescope, 70 cm long, diameter of 3.6 mm, insertion canal 1.2 mm). *M. bovis* suspension was instilled through an inner catheter (0.8 mL inner volume) inserted within the canal of the endoscope followed by 1 mL of PBS to flush through the catheter. After being submitted to experimental challenge, the badgers were laid on their right side for recovery from the anaesthesia.

### 2.8. Examination Post-Mortem and Scoring of the Severity of the Lesions

Between twelve to thirteen weeks post-challenge, all the badgers in each study were euthanized under general anaesthesia and submitted to a detailed post-mortem examination by pathologists blinded to the animals’ treatment. One group of four animals housed in a pen were removed on each day, in random order, over the course of ten working days. Twenty-eight tissues were collected for *M. bovis* culture and for histological examination (lymph nodes–left and right where appropriate: parotid, mandibular, retropharyngeal, axillary chain, inguinal chain, popliteal, hepatic, mesenteric, anterior mediastinal, bronchial, posterior mediastinal, and tonsils; lung lobes–left and right where appropriate: cranial, caudal, middle, accessory; organs: spleen, liver, kidneys; pleura/mediastinum, salivary gland, and fat). Complete lungs were immersed in buffered formalin and sliced to explore the presence of visible lesions in the parenchyma.

The severity of the visible TB lesions was described in a standard manner and scored using criteria established prior to the first experiment. All data were recorded in a purpose-built database for automatic calculation of lesion scores. The level of tuberculous disease was summarized by calculating a Disease Burden Score (DBS) [18] once the presence of *M. bovis* was confirmed by culture or by Ziehl Nielsen (ZN) staining in the tissues and when the severity of microscopic lesions was determined by histology.

### 2.9. Bacterial Culture

Clinical samples (tracheal aspirates, laryngeal swabs and rectal swabs) were taken from badgers under anaesthesia two weeks following vaccination and then every two to three weeks until challenge to enable detection of BCG excretion by culture. Collection of clinical samples post-vaccination only took place for vaccinated animals, not for the controls. Clinical samples were taken from two weeks post-challenge until post-mortem and submitted for culture to detect the excretion of *M. bovis* by all (vaccinated and control) badgers, as previously described in [10].

### 2.10. Histology

Tissues were submitted to histopathology after fixation by immersion in buffered formalin. Fixed tissues were blocked into cassettes (up to three tissues, of different types, were inserted per wax block) and embedded in paraffin wax. Serial sections four microns thick were cut and stained with haematoxylin and eosin stain, and with ZN stain. The severity of granuloma based on their cell structure and number of AF bacilli was scored as previously described in [10].

### 2.11. Immune Responses

Every 2–3 weeks, blood was collected under general anaesthesia to monitor peripheral immune responses by measuring the frequency of peripheral blood mononuclear cells (PBMC) producing IFNγ by enzyme-linked immune absorbent spot (ELISpot) assay [19] and using an IGRA as previously described in [15] to measure the abundance of IFNγ released in culture. Antigens used to stimulate PBMC cultures in duplicate wells were those used for the routine diagnosis of TB in humans and animals; namely, purified-protein derivative (PPD)B and PPDA (APHA Weybridge antigens). Concanavalin-A (ConA) was included in the ELISpot assay and IGRA as a positive control (mitogen) for PBMC ability to produce IFNγ.

### 2.12. Statistics

For analysis, ELISpot responses of PBMCs after stimulation by PPDB and PPDA antigens were log transformed to make their variance more uniform, using the transformation of raw data log_10_(*x* + 1), which was equivalent to log_10_(*x* + 5) in units of spot forming units/10^6^ cells. Transformation of the data was applied before duplicate measurements for each antigen were averaged. IGRA responses were also log transformed for the same reasons using log_10_(*x* − 0.047). All observed values were > 0.047, but many were close to that minimum value, and preparatory analysis found that the lowest observed values should be reduced to near zero before taking their logarithm to minimize skewness of the distribution of values after transformation. There were some inconsistent zero and low values from ELISpot, so observations were treated as missing if PPDB or PPDA values were <1.1 after transformation and averaging, while observations at other time points were consistently >1.7. One observation from VES9 at day 14, one observation from VES10 at day 77, and four observations from VES10 at day 91 were interpreted as missing by these criteria. In most cases, both PPDB and PPDA values were interpreted as missing. The one exception to this was the observation from VES10 at day 77, for which PPDB was interpreted as missing but not PPDA.

Four measurements by ELISpot and IGRA were taken between vaccination and challenge: in VES9 at days 14, 28, 70, and 91 after vaccination; in VES10 at days 28, 49, 77, and 91. Measures tended to peak early and slowly decrease over each of these periods, but the change was small relative to the variance at each time point. For this reason, and since the average of the four time points was expected to be a better summary value than a single observation, indicator values were calculated by taking the two means across the four time points. The averages for each antigen between vaccination and challenge were referred to as PPDBm and PPDAm.

The measure of disease, DBS, was derived from four measures of tuberculous disease as described by [18]: (1) a visible lesion score (VLS) based on the size and distribution of visible lesions once the presence of *M. bovis* was confirmed by culture or by ZN staining of the tissues; (2) a tissue count (the number of tissues with TB lesions confirmed by culture or ZN staining); (3) a granuloma score derived from H & E stained, formalin-fixed tissue; (4) the total surface area (in mm^2^) of lung with visible lesions estimated at post-mortem. The calculation of DBS included the transformation and standardization of each of the four component measures to zero mean with unit variance among observations on control badgers accumulated across multiple studies subjected to equivalent challenge and necropsy protocols, before calculating DBS for each badger as the sum of the four components, as described in [18].

The primary comparison of each study was to compare DBS between treatments, taking account of any other factors that potentially had a significant effect, including sex, weight at the time of vaccination, and group effects as identified by the pens occupied by badgers. Sex and weight had been identified as significant factors in a previous, larger preparatory analysis [18]. However, factors apart from treatment were excluded because they made no improvement to linear models of the individual studies. In VES9, the comparisons of two orthogonal contrasts were planned: the contrast between the control treatment and both vaccination treatments, and the contrast between the high and low dose vaccination treatments. In VES10, the primary contrast was between the control and vaccine bait treatments. Two comparisons were made: (1) including all badgers in the vaccine bait treatment, whether they consumed bait or not (evaluation based on intention to treat); and (2) excluding badgers that refused bait (evaluation based on exposure to vaccine in bait). Further analysis, using ANOVA and linear regression, was combined for VES9 and VES10, since both studies had similar designs and the control treatments and manual vaccination treatments matched across them.

Linear regression analysis was applied to estimate the dependence of DBS on PPDBm, PPDAm, and treatment. Other covariates including sex, weight at the time of vaccination, and group effects as identified by the pens occupied by badgers were considered for inclusion in models, but only sex was included because other covariates did not improve model fit. In these analyses, which were primarily concerned with the relationship between protection and immunology after vaccination, badgers that refused vaccine bait were treated as equivalent to additional controls.

Survival curve analysis for the *M. bovis* excretion data was performed by the Log-rank (Mantel-Cox) test using GraphPad Prism version 7.00 for Windows, GraphPad Software, La Jolla, CA, USA, www.graphpad.com.

## 3. Results

### 3.1. Vaccine Doses

The average CFU/mL of the two bioreactor-grown *M. bovis* BCG batches used for vaccination in studies VES9 & VES10 is presented in Table 1, for the pooled harvests of each BCG batch and the final concentrated suspensions. These resulted in doses of BCG delivered to each treatment group in each experiment shown in Table 2. In VES10, 18/20 badgers offered bait containing vaccine consumed it. The two badgers that refused bait were in different groups. In addition, two badgers had to be removed from VES10 for welfare reasons unrelated to treatment. Both were in the manual vaccination treatment, and one was in the same pen as a badger that refused vaccine bait.

### 3.2. Vaccine Efficacy as Determined by the Disease Burden Score (DBS)

Table 3 and Table 4 show the values of DBS for each badger in VES9 and VES10 respectively, along with the raw values before transformation of each of the four component measures. The VLS and granuloma score for each individual tissue for each badger in VES9 and VES10 are provided as Supplementary Tables S1 and S2, respectively.

The planned comparisons in VES9 found that average DBS was significantly reduced in badgers vaccinated manually, i.e., vaccine provided protection, but there was no significant difference between the protection from vaccine delivered at low dose and high dose (Table 5). VES10 confirmed that DBS was again significantly reduced in badgers vaccinated manually. However, DBS was not significantly affected by bait vaccination, whether or not badgers that refused bait were included in the comparison.

**Figure 1:**
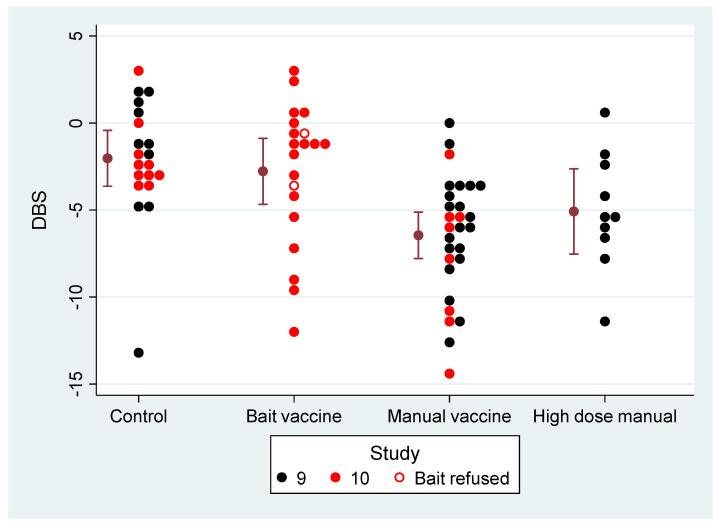
Disease Burden Score (DBS) plotted for each of the different treatments across VES9 (black dots) and VES10 (red dots). Controls were unvaccinated. The remaining badgers were either vaccinated by voluntary consumption of Bacillus of Calmette and Guérin (BCG) in bait (Bait vaccine, open dots refused bait) or by manual vaccination with 2.0 × 10^8^ colony forming units (CFU) (VES9) or 8.4 × 10^8^ CFU (VES10) BCG (plotted collectively as ‘Manual vaccine’) or with a higher dose (1.2 × 10^9^ CFU) of BCG in VES9 only (plotted as ‘High dose manual’). Bars indicate mean with its 95% confidence interval.

Figure 1 shows the DBS values from both studies, separated by treatment (control, bait vaccination, manual vaccination). The control and ‘Manual vaccine’ treatments were common to VES9 and VES10. The distribution of DBS values for these two treatments intermingled between the studies, showing that the two studies were highly reproducible in terms of challenge with *M. bovis* and manual vaccination with BCG. As suggested by visual comparison of observations in the control and manual vaccination treatments, analysis of variance found no evidence of a significant difference in DBS between VES9 and VES10. Therefore, we treated all the groups from VES9 and VES10 as if they were in a single large study. Observations from VES9 and VES10 were combined to provide more power to investigate treatment effects.

Combining the observations of VES9 and VES10, and assuming the manual vaccination treatments had equivalent effects to each other, we estimated the effect of manual vaccination with a narrower confidence interval (compare Table 6 with Table 5). The three manual vaccination treatments across the two studies did not significantly differ from each other (Table 6). The effect of bait vaccine relative to controls was still not statistically significant after pooling the studies. The difference between sexes was similar to the difference reported from previous studies [18], although its significance here was marginal (*P* = 0.06).

### 3.3. Excreted BCG or M. bovis within Clinical Samples

Clinical cultures were sown onto six 7H11 slopes at 2, 4, 10, and 13 weeks post-vaccination in both studies, and at 2, 5, 8, and 12 weeks post-challenge for culture of BCG and *M. bovis* in VES9 and at 2, 4, 7, 12, and 13 weeks post-challenge in VES10. Results are shown in Table 7; Table 8 for VES9 and in Table 9 only for VES10, as no BCG was found in clinical samples in VES10. BCG was excreted into the larynx and trachea post-vaccination, but not into faeces in VES9 (Table 7).

**Figure 2:**
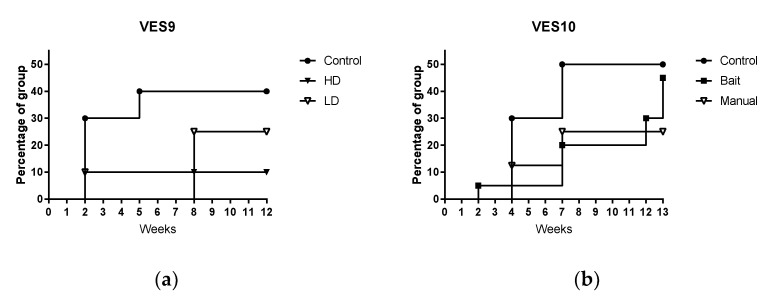
Survival curves showing the percentage of each treatment group in study VES9 (**a**) and study VES10 (**b**) excreting *M. bovis* in any clinical sample at the time that the first positive culture was produced. Controls = unvaccinated badgers. HD = manual vaccination with 1.2 × 10^9^ colony forming units (CFU) of Bacillus of Calmette and Guérin (BCG). LD = manual vaccination with 2.0 × 10^8^ CFU BCG. Bait = vaccinated with 8.6 × 10^8^ CFU BCG by voluntary consumption of BCG in bait. Manual = manual vaccination with 8.4 × 10^8^ CFU BCG.

*M. bovis* was excreted, mostly in the tracheal mucus, and without any initial clear pattern of differences between manual vaccinated and controls (Table 8 and Table 9). Figure 2 presents the excretion data for both experiments as survival curves. *M. bovis* excretion tended to occur in a greater proportion of control badgers and at an earlier time point than vaccinated badgers in both studies. However, when these data were subjected to statistical analysis (Log-rank (Mantel–Cox) test), no significant difference was found between the curves in either VES9 (*p* = 0.2299) or VES10 (*p* = 0.5104).

### 3.4. Immune Responses

**Figure 3:**
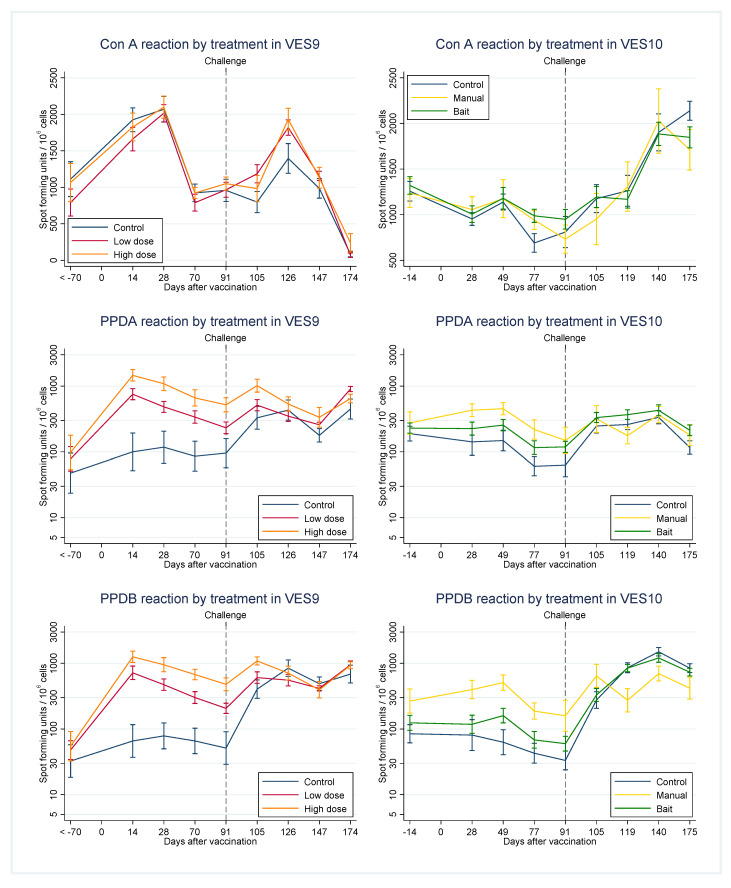
Interferon-gamma (IFNγ) direct enzyme-linked immune absorbent spot (ELISpot) assay results for VES9 left and VES10 right, for each of the stimulations shown (ConA = concanavalin A. Error bars are standard errors of the means.

**Figure 4:**
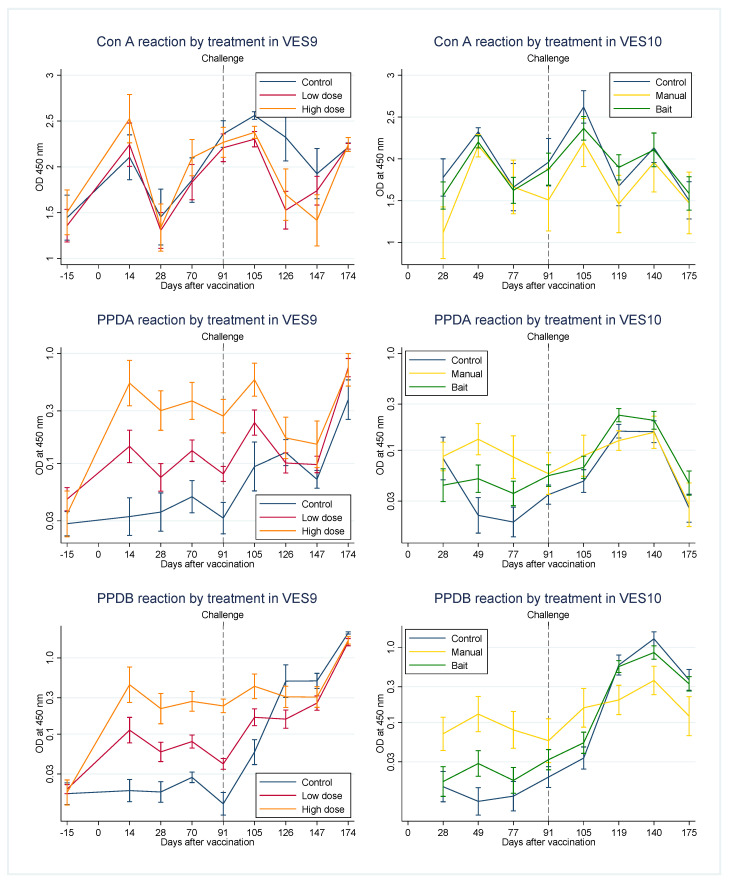
Peripheral blood mononuclear cells (PBMC) interferon-gamma (IFNγ) enzyme-linked immunosorbent assay (ELISA) (IGRA) for VES9 left and VES10 right, for each of the stimulations shown (ConA = concanavalin A). Error bars are standard errors of the means.

Cellular immune responses were assessed over the time course of VES9 and 10 by IFNγ direct ELISpot (Figure 3) and IFNγ enzyme-linked immunosorbent assay (ELISA) (IGRA) from stimulated cultures of PBMCs (Figure 4). The ELISpot response of PBMCs to ConA fluctuated throughout the study, dipping in particular on the day of post-mortem in VES9, as we have observed in other VES studies [10]. Importantly however, this did not prevent the response of PBMCs to antigens. The IGRA response to ConA was less variable in general over time compared to the ELISpot. ConA responses did not differ significantly between treatment groups at any time point in either study or in either assay. Raw data from ELISpot and IGRA in VES9 and VES10 are provided as Supplementary Tables S3 and S4, respectively.

Antigen-specific responses appeared broadly comparable between assays in each study. Responses to both PPDA and PPDB between vaccination and challenge tended to be greater in badgers receiving manual vaccination than in other treatments (Figure 3 and Figure 4). On the other hand, responses to PPDB increased after challenge more in controls and the bait vaccination treatment. A similar but weaker change was observed after challenge in the response to PPDA. The net effect was that PPDA and PPDB responses after challenge were often similar across treatments.

**Figure 5:**
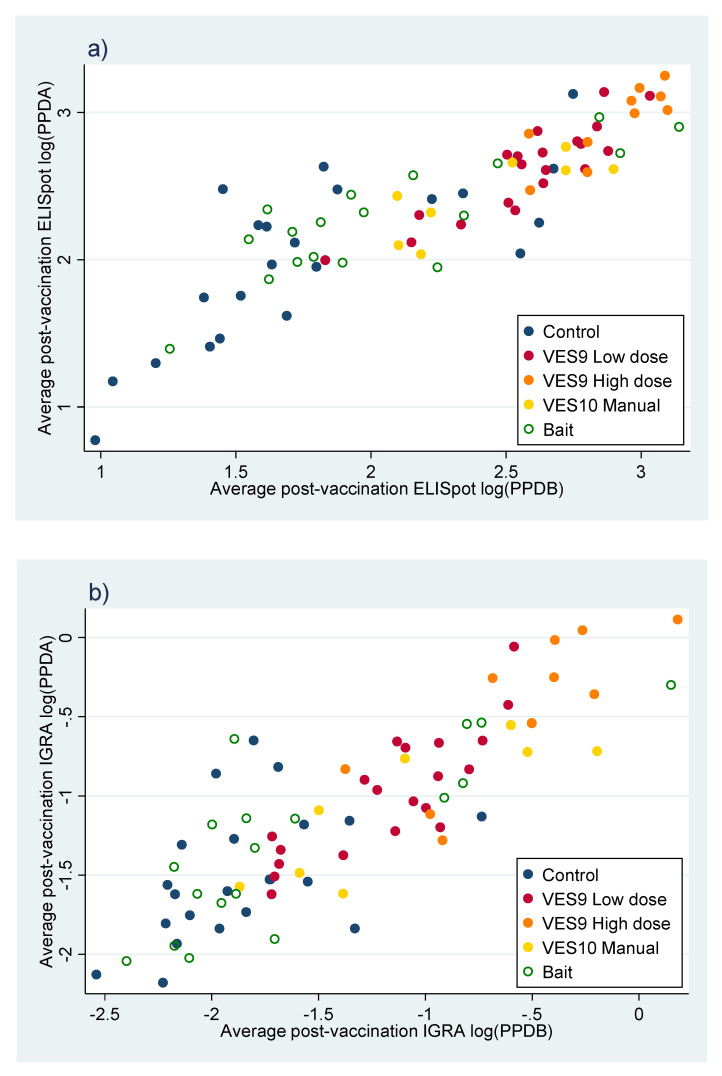
Measures of post-vaccination reaction to purified-protein derivative (PPD)A and PPDB measured by (**a**) enzyme-linked immune absorbent spot (ELISpot) assay and (**b**) IGRA, and transformed and summarised to PPDAm and PPDBm as described in the Methods. Each point summarises the post-vaccination state of one badger.

The PPDAm and PPDBm values, calculated as explained in the Methods from ELISpot and IGRA were used as summaries of the PPDA and PPDB responses post-vaccination up to challenge. The correlation between the two responses and their dependence on treatment is displayed as a point for each individual animal in Figure 5. Despite individual variation, a clear association could be seen between vaccine given manually and the pre-challenge IFNγ response, which even provided some discrimination between manual vaccination treatments. The IFNγ response in the Bait vaccination treatment mostly mingled with the control treatment but had a wide range that overlapped with the manual vaccination treatments more than the controls did, especially in the IGRA plot (Figure 5b). PPDAm and PPDBm were strongly correlated with each other, but linear discriminant analysis found that PPDBm was more important in differentiating the treatments (Table 10).

Comparing Figure 5a with Figure 5b also demonstrates that, despite the consistency between the ELISpot and IGRA observations (correlation coefficients: PPDAm 0.852, PPDBm 0.863), the different methods produce measures with different scaling. The resolution of ELISpot measures is relatively high at low values, separating control observations while bunching observations from vaccinated treatments, while IGRA measures have higher resolution at high values. Partly because they are both log-transformed, the two measures have similar range and variance, so a combined measure with more uniform resolution can easily be generated by adding together matching pairs of ELISpot and IGRA measurements, which will be referred to as ‘combined’ PPDAm and PPDBm.

**Figure 6:**
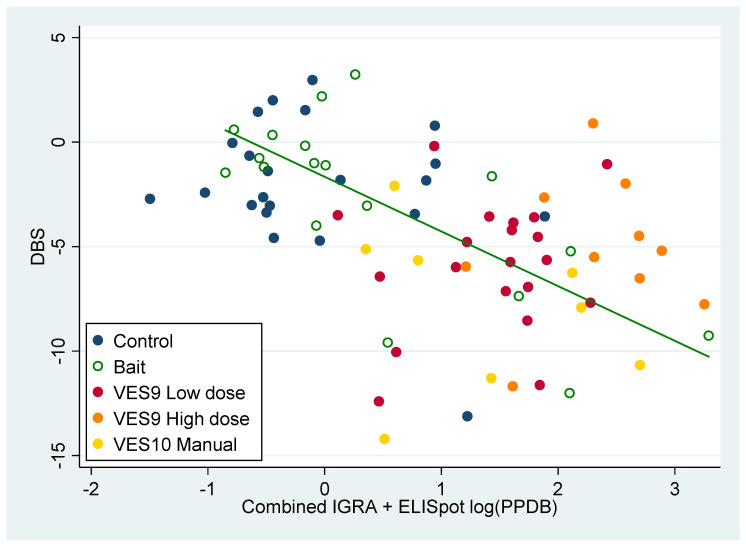
Disease Burden Score (DBS) of badgers post mortem plotted against purified-protein derivative (PPD)B reaction post-vaccination (PPDBm), from a combination of log-transformed enzyme-linked immune absorbent spot (ELISpot) assay and IGRA measures. Observations are classified into five treatment groups as in the legend. The line is fitted to the observations from the bait vaccination treatment only (*P* = 0.0003); although the trend roughly matches the differences between treatments, there was no significant trend related to PPDBm within the other treatments (Table 11).

Although the DBS values were visibly correlated overall with the combined PPDBm data (ELISpot plus IGRA) from both studies (Figure 6), PPDBm distinguishes treatments so well, the differences between treatments explain most of the relationship between DBS and PPDBm (see ‘manual vaccine v. control’ in Table 11). The exception is for the bait vaccination treatment, where there was a highly significant association between DBS and PPDBm for individual animals within the Bait vaccination treatment (Figure 6, ‘slope v. PPDBm’ in Table 11). Hence, where PPDBm was close to zero in bait vaccinated animals the DBS was similar to controls, but where higher values of PPDBm were observed in bait vaccinated animals the DBS was reduced compared to controls, indicating a degree of protection. In total, six badgers in the bait treatment group had higher combined PPDBm than the other twelve that ingested baits, and five of those six had lower DBS than other badgers in the group (Figure 6, see also Figure 1). The implication was that vaccine delivery varied within the Bait vaccine treatment, so that a minority of badgers (no more than six) were effectively vaccinated and protected against TB. The bait treatment group could in effect be classified by their PPDBm response into a majority (two-thirds) that responded to challenge like controls, and a minority (one-third) that responded like badgers receiving manual vaccination.

When the regression model included the combined PPDAm measure as well as PPDBm, the treatment differences remained broadly the same as with PPDBm alone, but some variation within treatments was associated with the difference between PPDBm and PPDAm (Table 12). DBS was generally negatively associated with PPDBm and positively associated with PPDAm, with an even stronger negative association with PPDBm in the bait vaccination treatment (Table 12). Hence, the magnitude of the post-vaccination IFNγ response before challenge was indicative of both successful vaccine delivery and some protection against challenge. The response to PPDB was the main discriminator between vaccine treatments and of vaccine delivery, while the response to PPDA relative to PPDB indicated variation within treatments. Note that a recent analysis of previous badger vaccination studies identified a similar relationship within treatments between DBS and PPDA response before vaccination [18]. However, it is notable that including PPDAm in the model had little impact on the estimated average treatment effects (compare Table 11 and Table 12).

## 4. Discussion

The studies presented here were planned to test the protective efficacy granted by oral bioreactor-grown BCG delivered by two different methods: (a) via the ingestion of a vaccine-bait product; and (b) by direct administration into the mouth under general anaesthesia. One key finding in the first study (VES9) was the ability of bioreactor-grown BCG to confer significant protection to badgers against *M. bovis* challenge via direct administration at a dose of at least 2.0 × 10^8^ CFU. The protection seen in VES9 with oral bioreactor-grown BCG was replicated in VES10 by the manual route. This result confirms what has been observed previously, that the oral route is suitable for protecting badgers against virulent *M. bovis* [10,11,20,21].

A second outcome of the studies was that BCG presented in voluntarily-consumed baits did not result in demonstrable protection at the group-level. However, detailed analysis suggested that vaccine was successfully delivered to and potentially protected no more than six out of 18 badgers that ingested baits. Whilst we do not know the crucial site(s) for immune induction following the administration of oral BCG, the results with manual vaccination suggest that delivery to the tonsils and underlying lymphoid-associated tissue of the oropharynx may be enough to induce protective immunity. This is supported by our recently published observations that following manual vaccination of badgers, significant quantities of live BCG were taken up by the tonsils, persisting in local lymphoid tissues of vaccinated badgers for at least eight weeks and inducing circulating IFNγ-producing mononuclear cells [22]. Whilst we cannot rule out a role for immune induction occurring through uptake of swallowed BCG via the intestines, we know uptake of live BCG to be less efficient when administered directly to the ileum [22]. Collectively, these data cause us to hypothesise that the limiting step for efficient bait vaccination is release of BCG into the mouth of the badger when bait vaccine is consumed. Previous unpublished observations of wild badgers exposed to the bagged bait used in this work showed that individuals spent on average 55 s (36 to 100 s, n = 5) consuming the bait (Defra research project SE3246), which is a relatively limited window of opportunity if indeed this is the most significant factor effecting the efficacy of oral bait vaccination of badgers. A similar situation was observed in the evaluation of an oral bait vaccine against rabies, where bait placed a constraint on the full release of the contained dose. Following 13 vaccine studies, it became clear that a higher dose of rabies vaccine was required in bait to achieve a similar level of protection to that administered manually [23]. This is significant, since the site of immune induction following oral rabies vaccination appears to be the oral cavity and not the intestine, at least based on where the vaccine replicates and can be detected [24]. Under a logical step-wise approach, future experiments would examine the delivery system in detail and allow for a re-design that would mimic, as closely as possible, the direct administration method. It is not unreasonable to hypothesise that in many cases the current bait design, which has a small volume of BCG in a relatively large bait-carrier mass [8], results in the mixing of the BCG in the bait during chewing with the result that most of it is swallowed.

We observed that antigen-specific IFNγ responses after vaccination were related to the degree of protection as expressed by DBS at the end of each study; protection being positively associated with the magnitude of the PPDB response. It was the general case, however, that PPDB responses were more discriminatory at the level of vaccine treatment than for individual animals. Study of the vaccination of cattle with BCG have concluded that post-vaccination antigen-specific IFNγ levels in whole blood are not always a reliable indicator of protection against a subsequent virulent challenge [25,26], although when PBMC were isolated from BCG-vaccinated calves immediately prior to *M. bovis* challenge and cultured in the presence of antigen and IL-2 to establish short-term T cell lines, the frequency of antigen-specific IFNγ-secreting memory cells did correlate with protection [27]. Beyond species differences, discrepancies between studies in respect to pre-challenge immune correlates of protection are likely to arise not only from the conditions of the assay (ex vivo versus cultured PBMC) but also the way the immunological data are treated before statistical analysis. For example, in [26], the authors looked to correlate the maximal quantity of IFNγ (expressed as ng/mL by IGRA) induced by vaccination with the degree of protection, whereas in [27] it was the frequency of IFNγ-secreting cells by ELIspot at a single time point. In our case, we not only took an average of all (four) time points post-vaccination but used a combination of ELISpot (frequency of IFNγ-producing cells per mL blood) and IGRA data, and log-transformed the data before analysis to improve the homogeneity of variance and to improve the linearity of the data so that the averaging could be beneficial. Of these differences, we suspect the averaging of the post-vaccination response was most important, since in a different cattle study the authors found that neither the levels of IFNγ detected by ELISA nor detected by ELISpot in individual vaccinated animals at the time of challenge correlated with subsequent resistance [25]. Note that the strong correlation found between average values from ELISpot and from IGRA in our study suggests that the data preparation substantially improved precision. This is an approach that should be considered in future vaccination-protection studies of TB and could even be applied retrospectively to existing data. In any case, the immune responses reported here are evidence of efficient exposure of badgers to the vaccine. In the bait vaccination treatment group, which overall did not achieve a statistically significant decrease in DBS, the pre-challenge immunology of around a third of badgers appeared equivalent to those of manually vaccinated badgers and with the level of protection observed in those individual badgers, which suggests some VES10 animals received a protective dose of BCG through bait delivery. This supports our contention that vaccination with the bait in the current design can give protection, but only to a minority of badgers that consume it.

## 5. Conclusions

We demonstrated repeatable protection against TB through the direct administration of at least 2.0 × 10^8^ colony forming units of BCG to the oral cavity of badgers. Vaccination via voluntary consumption of bait containing the same preparation of BCG did not result in demonstrable protection at the group-level, although there was evidence based on immune responses following vaccine bait delivery and pathology post mortem that a minority of badgers (no more than six from 18) were effectively vaccinated and protected against TB through bait delivery. The need to deliver oral BCG in the context of a palatable and environmentally robust bait appears to introduce such variation in BCG delivery to sites of immune induction in the badger as to render experimental studies variable and inconsistent. This currently poses the biggest impediment to the introduction of a robust oral bait vaccine against TB in badgers in the UK.

## Figures and Tables

**Table 1:** The average CFU/mL of *M. bovis* Bacillus of Calmette and Guérin (BCG) used for vaccination in studies VES9 & VES10 for the pooled harvest for each BCG batch and the final, concentrated, suspension.

Batch	CFU/mL ± SD
VES9	
Harvest	1.04 × 10^9^ ± 1.35 × 10^8^
Final	4.64 × 10^9^ ± 2.20 × 10^8^
VES10	
Harvest	4.40 × 10^8^ ± 1.50 × 10^8^
Final	2.53 × 10^9^ ± 6.40 × 10^8^

**Table 2:** Details of treatment groups in the two experiments: mean vaccine doses and method of vaccination.

Study	BCG Dose (CFU)	Method of Vaccination	Number of Badgers
VES9	2.0 × 10^8^	Manual	20
	1.2 × 10^9^	Manual	10
	None	N/A	10
VES10	8.4 × 10^8^	Manual	8 (originally 10)
	8.6 × 10^8^	Bait	20
	None	Placebo bait ^1^	10

^1^ Bait prepared and presented in the same way as vaccine baits but containing no BCG.

**Table 3:** Burden of disease for each animal *post mortem* in VES9, as assessed by four different measures, as described above.

Treatment	Badger ID	VLS	Tissue Count	Granuloma Score	VL Surface Area ^1^ (mm^2^)	DBS ^2^
Control	NEC-077	10	19	18	300	0.79
	NEC-110	11	13	14	678	2
	NEC-261	5	6	10	20	−4.58
	NEC-293	11	10	14	474	1.53
	NEC-338	5	5	6	190	−4.71
	NEC-541	5	13	11	269	−1.81
	NEC-5591	7	8	10	248	−1.03
	NEC-606	8	8	15	153	−1.38
	NEC-616	0	3	1	2	−13.12
	NEC-9353	8	13	16	602	1.45
HD ^3^	NEC-001	4	2	4	180	−5.19
	NEC-049	14	11	20	91	0.9
	NEC-1081	3	6	9	13	−5.95
	NEC-113	2	8	6	61	−5.5
	NEC-326	6	7	14	84	−2.65
	NEC-351	1	3	6	22	−7.76
	NEC-5012	1	5	0	4	−11.68
	NEC-514	3	4	8	14	−6.51
	NEC-782	4	4	13	17	−4.49
	NEC-808	7	8	16	52	−1.98
LD ^4^	NEC-035	6	8	14	345	−1.05
	NEC-041	1	4	9	1	−7.68
	NEC-1856	1	6	11	7	−7.13
	NEC-257	4	2	5	140	−5.64
	NEC-2576	1	4	8	75	−6.43
	NEC-260	7	6	12	13	−4.21
	NEC-315	5	7	10	90	−3.5
	NEC-325	4	6	16	4	−4.78
	NEC-337	9	8	13	274	−0.19
	NEC-349	0	2	3	0	−11.62
	NEC-371	4	4	7	45	−5.74
	NEC-373	6	6	7	220	−3.56
	NEC-524	6	3	6	114	−4.53
	NEC-622	6	7	9	117	−3.85
	NEC-7073	2	2	5	0	−8.54
	NEC-772	1	2	2	0	−12.41
	NEC-774	4	8	6	236	−3.6
	NEC-817	1	3	7	145	−5.98
	NEC-889	2	2	4	2	−10.05
	NEC-9262	3	2	5	27	−6.93

^1^ Surface area may be zero if visible lesions were not found in the lung, only in other tissues. ^2^ DBS: Disease Burden Score, calculated from the first four variables shown as per [18]. ^3^ HD: Manual vaccination with 1.2 × 10^9^ CFU BCG. ^4^ LD: Manual vaccination with 2.0 × 10^8^ CFU BCG.

**Table 4:** Burden of disease for each animal *post mortem* in VES10, as assessed by four different measures, as described above.

Treatment	Badger ID	VLS	Tissue Count	Granuloma Score	VL Surface Area ^1^ (mm^2^)	DBS ^2^
Bait	NEC-1589	5	8	14	350	−0.76
	NEC-1624	9	7	13	221	−1.01
	NEC-2298	6	6	9	468	−1.18
	NEC-2863	5	4	11	62	−3.37
	NEC-2885	1	2	3	4	−9.59
	NEC-289	17	11	23	298	3.24
	NEC-327	6	5	7	839	−1.46
	NEC-3587	1	3	8	42	−7.37
	NEC-3856	10	10	15	121	−0.17
	NEC-5537	14	8	17	537	2.19
	NEC-5593	9	9	12	470	0.34
	NEC-5790	0	2	2	3	−12.01
	NEC-6283	2	2	4	4	−9.26
	NEC-6773	8	7	13	246	−1.11
	NEC-768	4	11	12	185	−3.04
	NEC-7879	1	4	8	232	−5.22
	NEC-833	9	6	8	354	−1.63
	NEC-8375	8	4	6	178	−3.99
	NEC-859	10	9	18	211	0.6
	NEC-9836	11	8	16	196	−0.65
Control ^3^	NEC-2560	7	6	9	82	−3.56
	NEC-3087	8	7	12	89	−2.71
	NEC-4042	5	6	10	260	−3.04
	NEC-4280	6	9	13	66	−3.01
	NEC-523	4	7	12	75	−2.64
	NEC-7066	9	9	13	144	−1.83
	NEC-7368	4	9	18	11	−3.44
	NEC-7525	16	13	18	570	2.97
	NEC-8072	3	7	14	195	−2.42
	NEC-882	10	7	16	293	−0.04
Manual	NEC-263	0	2	1	0	−14.2
	NEC-310	11	7	10	86	−2.09
	NEC-3620	3	4	7	36	−6.25
	NEC-4028	0	2	4	0	−11.29
	NEC-595	1	2	2	0	−10.67
	NEC-6109	5	6	4	36	−5.65
	NEC-7268	1	8	15	12	−5.12
	NEC-372	4	3	4	4	−7.91

^1^ Surface area may be zero if visible lesions were not found in the lung, only in other tissues. ^2^ DBS: Disease Burden Score, calculated from the first four variables shown as per [18]. ^3^ Bait prepared and presented in the same way as vaccine baits but containing no BCG.

**Table 5:** Planned comparisons of Disease Burden Score (DBS) between treatments.

Study	Comparison	Contrast	Std. Error	95% Confidence Interval ^1^
VES9	Vaccine vs. Control	−3.39	1.34	−6.10 to −0.68
	High dose vaccine vs. Low dose	0.79	1.4	−2.04 to 3.62
VES10	Bait vaccine (all) vs. Control	−0.8	1.4	−3.64 to 2.04
	Bait vaccine (consumed) vs. Control	−0.89	1.46	−3.85 to 2.08
	Manual vaccine vs Control	−5.93	1.75	−9.50 to −2.36

^1^ Significant where the interval does not span zero.

**Table 6:** Treatment and sex effects from an analysis of variance of Disease Burden Score (DBS) across VES9 and VES10. Badgers that refused vaccine bait have been excluded from this analysis.

Source	Value	Std. Error	P	95% Confidence Interval
Treatments				
Bait vaccine vs. control	−1.02	1.17	0.39	−3.34 to 1.31
Manual vacc. vs. control	−4.23	0.99	<0.001	−6.22 to −2.25
High dose vs. Low dose VES9	0.95	1.38	0.49	−1.81 to 3.70
VES10 vs. Low dose VES9	−1.91	1.49	0.21	−4.88 to 1.06
Sex				
Male vs. female	1.6	0.85	0.06	−0.10 to 3.30

**Table 7:** Clinical samples from VES9 that yielded a positive Bacillus of Calmette and Guérin (BCG) culture ^1^, derived from badgers that received the higher dose of BCG manually ^2^.

Badger ID	Weeks after Vaccination			
	2 weeks	4 weeks	10 weeks	13 weeks ^3^
NEC-001	LS	LS	LS	
NEC-049	TA	LS, TA		
NEC-1081				
NEC-113				
NEC-326	LS	LS		
NEC-351		LS	TA	
NEC-5012				
NEC-514	LS	LS, TA		
NEC-782	LS			
NEC-808				

^1^ LS = laryngeal swab; TA = tracheal aspirate. ^2^ No BCG excretion was detected from control badgers nor those vaccinated manually with the lower dose of BCG in VES9. ^3^ Day of challenge with *M. bovis*–no BCG was cultured from any clinical sample on this occasion.

**Table 8:** Clinical samples from VES9 that yielded a positive *M. bovis* culture ^1^.

Treatment	Badger ID	Weeks after Challenge with *M. bovis*			
		2 weeks	5 weeks	8 weeks	12 weeks ^2^
Control	NEC-077		TA		
	NEC-110				
	NEC-261				
	NEC-293				
	NEC-338				
	NEC-541	LS, TA			
	NEC-5591				
	NEC-606	TA			LS, TA
	NEC-616				
HD ^3^	NEC-9353	TA			
	NEC-001				
	NEC-049				
	NEC-1081				
	NEC-113			TA	
	NEC-326				
	NEC-351				
	NEC-5012				
	NEC-514				
	NEC-782				
	NEC-808				
LD ^4^	NEC-035			LS, TA	
	NEC-041				
	NEC-1856				
	NEC-257	TA			
	NEC-2576				
	NEC-260				
	NEC-315				
	NEC-325				
	NEC-337			LS, TA	
	NEC-349				
	NEC-371				
	NEC-373	LS, TA			TA
	NEC-524				
	NEC-622				
	NEC-7073				
	NEC-772				
	NEC-774			LS	
	NEC-817				
	NEC-889				
	NEC-9262				

^1^ LS = laryngeal swab; TA = tracheal aspirate. ^2^ Day of post-mortem. ^3^ HD: Manual vaccination with 1.2 × 10^9^ CFU BCG. ^4^ LD: Manual vaccination with 2.0 x 10^8^ CFU BCG.

**Table 9:** Clinical samples from VES10 that yielded a positive *M. bovis* culture ^1^.

Treatment	Badger ID	Weeks after Challenge with *M. bovis*				
		2 weeks	4 weeks	7 weeks	12 weeks	13 weeks ^2^
Bait	NEC-1589			TA		
	NEC-1624	RS			LS	
	NEC-2298					
	NEC-2863					
	NEC-2885					
	NEC-289				TS, TA	
	NEC-327					
	NEC-3587					
	NEC-3856					
	NEC-5537			LS, TA	LS, TA	
	NEC-5593					LS
	NEC-5790					
	NEC-6283					
	NEC-6773			LS, TA		
	NEC-768					F, RS, TA
	NEC-7879					
	NEC-833					
	NEC-8375					
	NEC-859					TS, TA
	NEC-9836				LS	
Control ^3^	NEC-2560					
	NEC-3087					
	NEC-4042		TA			
	NEC-4280					
	NEC-523					
	NEC-7066		TA	LS, TA	LS, F, TA	
	NEC-7368			TA		
	NEC-7525			LS	LS	
	NEC-8072		LS	TA		
	NEC-882					
Manual	NEC-263					
	NEC-310					
	NEC-3620			TA	TA	
	NEC-4028					
	NEC-595					
	NEC-6109					
	NEC-7268		LS	TA		
	NEC-372					

^1^ TA = tracheal aspirate; RS = rectal swab; LS = laryngeal swab; TS =tracheal swab; F = faecal swab. ^2^ Day of post-mortem. ^3^ Bait prepared and presented in the same way as vaccine baits but containing no BCG.

**Table 10:** Standardised loadings of PPDAm and PPDBm in the primary discriminant from a linear discriminant analysis between treatment groups in VES9 and VES10 (Stata 15 ‘candisc’ command). The magnitude of these loadings can be understood as formal measures of the relative contributions made to differentiating vaccination treatments, i.e., PPDBm was the main measure of treatment effect on immunology, especially when measured by enzyme-linked immune absorbent spot (ELISpot) assay, whereas the association of PPDAm with treatments was mainly due to its correlation with PPDBm. The data used in this analysis are displayed in Figure 5.

Method	PPDAm Loading	PPDBm Loading
ELISpot	0.017	0.987
IGRA	0.217	0.830

**Table 11:** Analysis of variance of Disease Burden Score (DBS) using combined PPDBm as the only measure of post-vaccination immunology.

Parameter	Estimate	Std. Err.	*t* Ratio	Prob > *t*	95% Confidence Interval
Manual vaccine v. Control	−4.54	1.32	−3.45	0.007	−7.16 to −1.92
Bait					
Intercept v. Control ^1^	0.09	1.10	0.08	0.94	−2.10 to 2.28
Slope v. PPDBm ^2^	−2.71	0.87	−3.11	0.003	−4.44 to −0.98
General slope v. PPDBm ^3^	0.14	0.55	0.26	0.80	−0.95 to 1.24
Male v. Female	1.49	0.80	1.87	0.07	−0.10 to 3.08

^1^ The difference between Bait vaccination and Control treatments at combined PPDBm = 0. ^2^ Slope of the regression of DBS against combined PPDBm within the Bait vaccination treatment. ^3^ Slope of a regression of DBS against combined PPDBm within Control and Manual vaccination treatments.

**Table 12:** Analysis of variance of Disease Burden Score (DBS) using combined PPDAm as well as PPDBm to measure post-vaccination immunology.

Parameter	Estimate	Std. Err.	*t* Ratio	Prob > *t*	95% Confidence Interval
Manual vaccine v. Control	−4.24	1.22	−3.45	0.001	−6.67 to −1.81
Bait					
Intercept v. Control ^1^	−0.18	1.02	−0.18	0.86	−2.21 to 1.85
Slope v. PPDBm ^2^	−2.32	0.81	−2.86	0.006	−3.94 to −0.70
General slope v. PPDAm ^3^	2.40	0.66	3.63	0.001	1.08 to 3.72
General slope v. PPDBm ^3^	−1.82	0.74	−2.45	0.02	−3.30 to −0.34
Male v. Female	1.23	0.74	1.66	0.10	−0.25 to 3.08

^1^ The difference between Bait vaccination and Control treatments at combined PPDBm = 0. ^2^ Slope of the regression of DBS against combined PPDBm within the Bait vaccination treatment only. ^3^ Slope of a regression of DBS against combined PPDAm or PPDBm within all treatments, i.e., fitting parallel lines with the same slope within each treatment.

## Supplementary Materials

The following are available online at https://www.mdpi.com/1999-4923/12/8/782/s1, Tables S1 and S2: The VLS and granuloma score for each individual tissue for each badger in VES9 and VES10, respectively. Tables S3 and S4: Raw data from ELISpot and IGRA in VES9 and VES10, respectively.
